# Acute treatment of ischemic stroke in 19 Sámi language administrative municipalities in the rural inland of Northern Sweden

**DOI:** 10.3389/fstro.2026.1759945

**Published:** 2026-07-01

**Authors:** Elisabeth Ronne Engström, Christoffer Nyberg, Mia von Euler

**Affiliations:** 1 Division of Neurosurgery, Department of Medical Sciences, Uppsala University, Uppsala, Sweden; 2 Division of Neuroradiology, Department of Surgical Sciences, Uppsala University, Uppsala, Sweden; 3 Sophiahemmet University, Stockholm, Sweden

**Keywords:** acute ischemic stroke, health care information, reperfusion treatment, rural, Sámi

## Abstract

**Objects:**

The effective treatment of acute stroke requires a series of events, including the recognition of stroke symptoms, following known action plans, and the rapid transfer to a hospital with sufficient medical competence. This is challenging in rural areas with long distances between hospitals with emergency departments. We studied the availability and effectiveness of acute stroke treatment in the most rural parts of northern Sweden. Some of the population in the study area belong to the Sámi, Sweden's indigenous people. Our aim was to study the yearly incidence of ischemic stroke, time window from start of symptoms to arrival at first hospital, and rate of revascularization treatment in the study population and compare to the rest of Sweden.

**Methods:**

Statistics Sweden defined the study group which was 142,127 individuals registered as living in the study area sometimes during 2019–2021. We used data from the National Board of Health and Welfare (NBHWF) regarding incidence and from the Swedish Stroke Register (Riksstroke, RS), for time windows and treatments. The study area was compared with the Swedish national data. Transfer times between municipalities, first hospitals, and thrombectomy centers were assessed using open data.

**Results:**

The incidence of ischemic stroke in the study group was 280/100.000/year which was significantly higher than national data for the same age group. 1,153 stroke incidents were registered in RS. In the study group 21.1% arrived at the first hospital >24 h from start of symptoms compared to 4.5% in national data. Three percent were treated with thrombectomy and 11.1% with thrombolysis. The numbers were small but those with the lowest percentage of arrival < 3 h, and of reperfusion treatment, all had the longest distances to the first hospital. 26.1 and 17.9% in two municipalities had an unknown time window from start of symptoms. A majority of the population would have shorter distances to thrombectomy centers outside Sweden.

**Conclusion:**

Our data shows a higher incidence of ischemic stroke in the study area in the rural northern Sweden. Persons with stroke in this area are unlikely to receive acute care in accordance with the Swedish national stroke guidelines.

## Introduction

In Sweden, much of the population live in the far south and in cities. However, since the rural areas are large geographically, a substantial number of people live there as well. Obviously, highly specialized medical care must be concentrated to a few centers, but there are several conditions that require immediate attention and treatment. This means that a substantial part of the population, those living in rural areas, may be denied important treatments. We wanted to study these aspects regarding acute stroke.

Health care in Sweden is gradually getting more digitalized. There is a website, www.1177.se, which has become the main hub for medical information. Through 1177 the residents can schedule health care visits, communicate with units in health care, that is, in primary care, hospitals, physiotherapy, etc. Most adult individuals can read their medical records through the website. 1177 also provides information about >100 medical conditions. Regarding “Stroke” there is extended information, and also a video, about symptoms and action plan. The information is available in Swedish.

However, Sweden has an indigenous people, the Sámi, that speak Swedish as well as varieties of the Sámi language, with either of the two languages as their first language. They have certain statutory rights, for example, to use their own language in contacts with the municipality society. Municipalities offering this are called “Sámi language administrative municipalities.” There are today 27 of such municipalities and 26 are located to northern Sweden. Our study area consisted of 19 of these municipalities in the most rural parts of northern Sweden.

The indigenous aspect was of interest since earlier studies have suggested increased incidence of cardio- and cerebro-vascular diseases in the Swedish Sámi group (Sjolander et al., 2008). Sweden does not register ethnicity and probably a minority of the population in the study area are known Sámi. However, due to historical factors (Lundmark, 2008), there is an unknown amount of the population that are Sámi descendants, which could have an effect both on genetics and lifestyle, and indirectly on health risk factors. We were also interested in the possible importance of appropriate information in correct languages about acute stroke. Nevertheless, the results apply to the whole adult population in the study area independent on background.

The aim was to study the whole adult population in a defined geographic area in northern rural Sweden, consisting of 19 Sámi language administrative municipalities, and compare it with the Swedish national population as a whole regarding:

- Yearly incidence of ischemic stroke, recorded in the national patient registers as ICD-10 code I63 (World Health Organization, International Classification of Diseases, Revision 10),
- Percentage of individuals with ischemic stroke that were registered in the Swedish Stroke Register “Riksstroke” (RS),
- Time window between start of symptoms until arrival at first hospital,
- Percentage of patients receiving reperfusion treatment with thrombolysis and/or thrombectomy.
- The capability for fast transfer of patients, for example, ambulance response times and distances between municipalities, first hospitals and thrombectomy centers.

## Materials and methods

### Data sources

The study was based on five data sources.

- Statistics Sweden for generating the study population and demographic data,
- National Board of Health and Welfare for data regarding time points, locations and diagnoses for inpatient and outpatient care periods,
- The Swedish stroke register “Riksstroke” regarding information about acute stroke treatment.
- Data from “Kolada” (www.kolada.se) for estimating response times for ambulances on emergency calls. This is an open database maintained in collaboration between the state and the Swedish Association of Local Authorities and Regions (SALAR).
- www.1177.se for health care information.

## Study population

**Figure 1:**
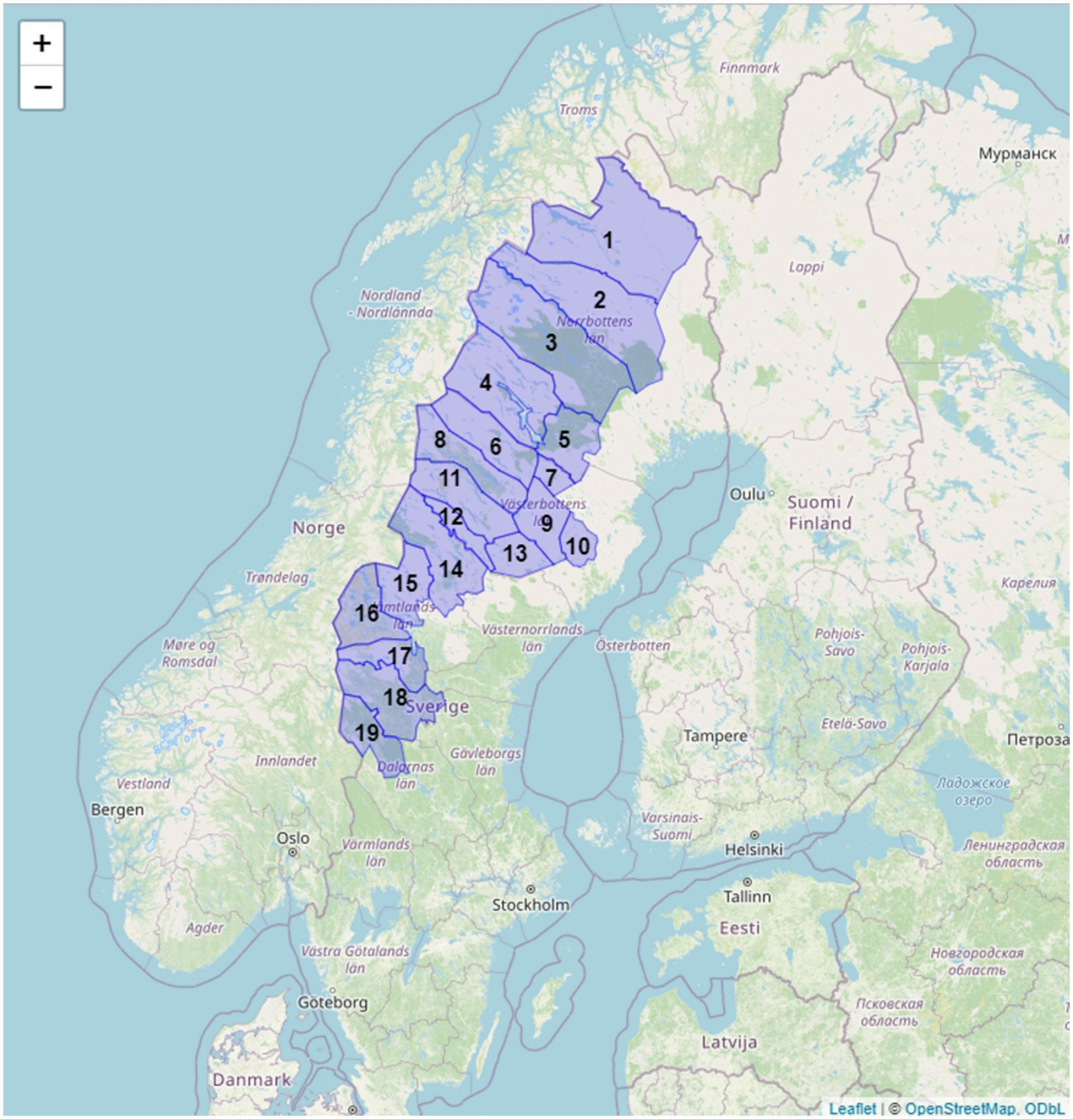
In this figure the study area is shown. This is the Sámi language administrative municipalities of 1. Kiruna, 2. Gällivare, 3. Jokkmokk, 4. Arjeplog, 5. Arvidsjaur, 6., Sorsele, 7. Malå, 8. Storuman, 9. Lycksele, 10. Vindeln, 11. Vilhelmina, 12. Dorotea, 13. Åsele, 14. Strömsund, 15. Krokom, 16. Åre, 17. Berg, 18, Härjedalen, 19. Älvdalen. The map shows that this area is placed in the northern rural part of Sweden, to a large extent along the border between Sweden and Norway.

The study population (*n* = 142,127) was defined as all individuals, ≥18 years old, who were registered as residents in the study area for any period of time during the years 2019 to 2021. The study area consisted of 19 Sámi language administrative municipalities located in the rural parts of northern inland Sweden (Figure 1). Seven municipalities were excluded since they were cities and/or located along the coastal highway and thus not considered rural by us. Age was defined as the age when the individual was first registered in the study area during the study period. The median age in the geographical area studied was 52 (IQR 33, 68) years, with 48% women and 52% men. The municipality of residence was defined as the first registered municipality in the study area, during the study period, but when evaluating the acute stroke treatment, municipality at the time of the incident was used.

## Stroke data

For estimating the incidence of ischemic stroke in the study group and comparing with open national data we used care episodes with the NBHWF main diagnoses of ICD-10 code I63. The incidence in the study area was compared to the incidence in the rest of Sweden. The same age group was selected from the open data but a close age matching was not done. The coverage of RS was calculated as the number of individuals registered in RS with main diagnoses I63 in NBHWF vs. the total number of individuals in the study area with main diagnoses I63 in NBHWF.

In analyzing the acute stroke treatment, we used RS data to determine time windows between start of symptoms, admission to first hospital, and reperfusion treatment with thrombectomy or thrombolysis. The results were compared to the yearly reports 2019–2021 published on the RS website (Riksstroke, 2021, 2022, 2020). Stroke incidents in the study group registered in RS but occurring outside the study area were excluded.

## Analyses of transfer

**Figure 2:**
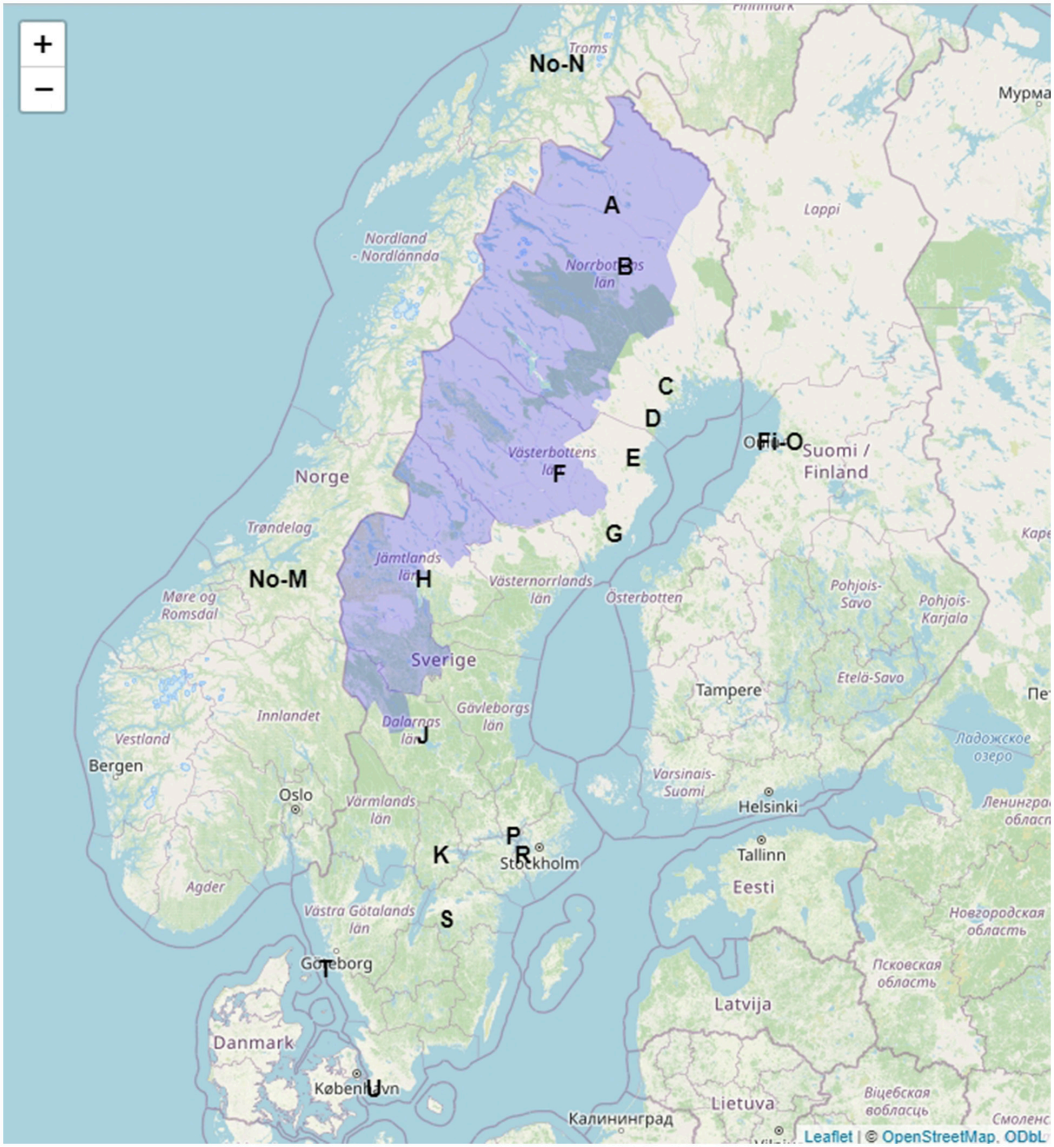
This figure shows the location of hospitals and of thrombectomy centres. A. Kiruna, B. Gällivare, C. Sunderbyn, D. Piteå, E. Skellefteå, F. Lycksele, G. Umeå, H. Östersund, J. Falun. Thrombectomy centers were in G. Umeå, K. Örebro, P. Uppsala, R. Stockholm, S. Linköping, T. Göteborg, U. Lund. Thrombectomy centers closest to the study area but outside Sweden were in Trondheim (No-M) and Tromsø (No-N) in Norway and Oulu in Finland (Fi-O).

In the study area, there are three hospitals. Patients were also admitted to hospitals located in the surrounding geographical area (Figure 2). The primary transport was usually by road ambulances, stationed in the local municipality. The distances between the respective municipality and the primary hospital refer to road transport. During the study period, thrombectomies were primarily performed at Umeå University hospital, but also in the University hospitals in Uppsala and Stockholm, both located in the south of Sweden. Thrombectomy usually required a secondary transfer. The secondary transports were done with helicopter or with road ambulance. The distances between primary hospitals and thrombectomy centers are shown both for airborne and road transfers based on open data (Himmera.com, 2025).

## Health care information

The website 1177.se was screened for information about stroke in the Sámi varieties by the first author. The results were checked by a person speaking North Sámi and South Sámi.

## Statistics

Analyses and visualization of data were done with RStudio, 2024.04.2, Posit Software, PBC, and Statistica 14.0 Cloud Software Group Inc, San Remo, CA, USA. Groups were compared with *t*-test and with Chi^2^. A *p*-value < 0.05 was considered significant.

## Ethical considerations

The Swedish ethical review authority granted permission for the study.

## Results

### Incidence of ischemic stroke

In the study group, 1,196 patients, or an average of 399 patients per year, had an ischemic stroke (I63) as the main diagnosis according to NBHWF. This resulted in an incidence of 281/100,000/year. We compared this with open data from the rest of Sweden for the same age group and during the same years. We found this to be 244/100,000/year which was significantly less than our study group (*p* < 0.006).

### Estimated coverage of RS

Of the 1,196 patients registered in NBHWF with I63 as the main diagnoses were 1,024 also registered in RS. This gave RS a coverage of 86%. Seventy patients in RS had the diagnosis I63 on lower levels. We compared those with main diagnosis of I63 in NBHWF that were and were not registered in RS. Stroke patients not registered in RS were significantly younger (mean 71.1 ± 15.5 vs. 75.5 ± 11.1 years, *p* < 0.00002). There was also a non-significant trend with a higher percentage of women in the group not registered in RS (49% vs. 44%, *p* = 0.16).

## Admission to first hospital and reperfusion treatment

**Figure 3:**
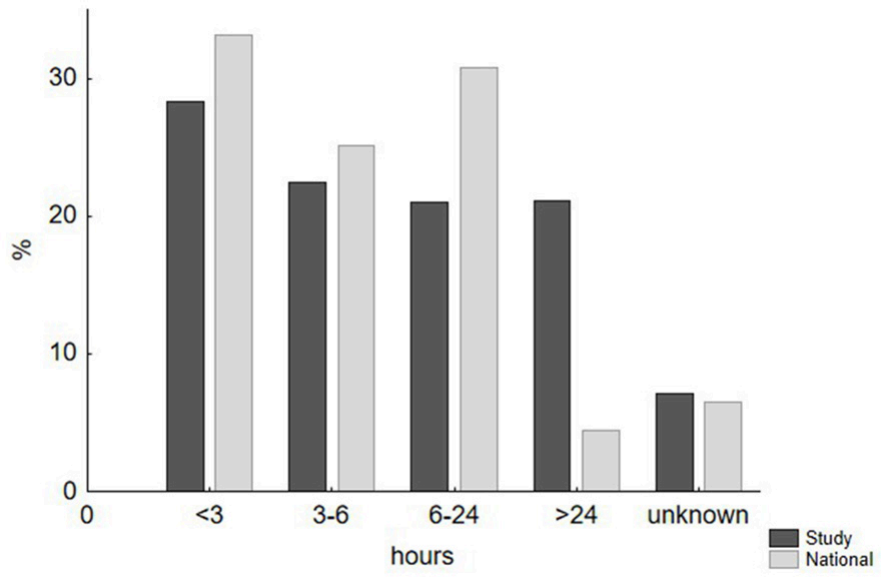
This figure shows the distribution of time windows from start of symptoms until admission to the first hospital. There was a much larger percentage of the study group that arrived >24 h after start of symptoms, compared to the national data.

For this analysis we used RS data from all the 1,153 incidents generated from the 1,094 individuals in RS. In 93 % the first contact was at the hospital in the municipality the patient belonged to geographically. We estimated the time window from start of symptoms to admission to the first hospital and found for the whole group that 28.4% arrived at hospital within 3 h, 22.5% between 3 and 6 h, 21.0% 6 and 24 h, 21.1% >24 h, and in 7.1% the time window was unknown. Figure 3 shows the time window for the study material and for national data during the same period. The delay in admission in the study group is illustrated in all the time windows, especially regarding admission >24 h after start of symptoms. Data for each municipality regarding time window < 3 h and “unknown” is shown in Table 1.

**Table 1:** Data from RS for each municipality.

Municipality	Stroke	Thrombectomy		Thrombolysis		Untreated		TW<3 h		TW unknown	
	*N*	*N*	%	*N*	%	*N*	%	*N*	%	*N*	%
Kiruna	147	2	1.4	18	12.2	127	86.4	46	31.3	7	4.8
Gällivare	118	3	2.5	16	13.6	99	83.9	40	33.9	7	5.9
Jokkmokk	23	1	4.3	5	21.7	17	73.9	9	39.1	1	4.3
Arjeplog	23	0	0.0	4	17.4	19	82.6	4	17.4	6	26.1
Arvidsjaur	56	0	0.0	8	14.3	48	85.7	9	16.1	10	17.9
Sorsele	22	0	0.0	3	13.6	19	86.4	5	22.7	0	0.0
Malå	29	2	6.9	4	13.8	23	79.3	11	37.9	0	0.0
Storuman	48	1	2.1	4	8.3	43	89.6	10	20.8	2	4.2
Lycksele	88	5	5.7	9	10.2	74	84.1	43	48.9	6	6.8
Vindeln	42	2	4.8	6	14.3	34	81.0	10	23.8	2	4.8
Vilhelmina	53	3	5.7	5	9.4	45	84.9	15	28.3	5	9.4
Dorotea	24	1	4.2	2	8.3	21	87.5	4	16.7	1	4.2
Åsele	14	2	14.3	3	21.4	9	64.3	6	42.9	0	0.0
Strömsund	95	3	3.2	11	11.6	81	85.3	18	18.9	9	9.5
Krokom	104	3	2.9	12	11.5	89	85.6	38	36.5	8	7.7
Åre	66	1	1.5	6	9.1	59	89.4	12	18.2	6	9.1
Berg	64	4	6.3	3	4.7	57	89.1	15	23.4	3	4.7
Härjedalen	77	0	0.0	6	7.8	71	92.2	16	20.8	6	7.8
Älvdalen	60	2	3.3	4	6.7	54	90.0	16	26.7	3	5.0

The numbers and percentages of stroke incidents, thrombectomy, thrombolysis, and untreated is shown. Time windows < 3 h and unknown, from start of symptoms to arrival to first hospital is also shown.

Table 1 shows the number of stroke incidents registered in RS for each municipality, and the number and ratio of reperfusion treatment. In the whole group 3.0% were treated with thrombectomy, 11.1% with thrombolysis and 85.8% had no treatment. Of the 75 patients already at a hospital when stroke symptoms started, 9.3% had a thrombectomy and 12.0% a thrombolysis. Thirty-two of reperfusion treatments through thrombectomy were done in Umeå, two in Uppsala and one in Stockholm.

For residents from four of the municipalities no thrombectomy was done. RS data contains information about why intravenous reperfusion treatment was not done. The most common reason was the time factor (33%), followed by “other” (32%), too mild symptoms (19%), other contraindications (15%), unknown (8%), and too severe symptoms (2%). There was no case in which lack of thrombolysis competence or failing emergency routines was considered the cause for not giving thrombolysis. The results from the study area were almost identical to the median national results during the study period, with 33%, 30%, 18%, 14%, 5%, and 2%, respectively.

## Transfer time

Road ambulances brought all the unconscious patients and 73.6% of all stroke incidents that were not already at the hospital.

Table 2 shows the mean road ambulance response time for each municipality (data from KOLADA.se). The table also shows the distance from the municipality center to the primary hospital. The four municipalities that had no thrombectomy done (Table 2) were also the four with longest distance to the primary hospital in Table 3.

**Table 2:** Open data (see section materials and methods) regarding ambulance response time on emergency calls, and the distance from each municipality center to the closest first hospital.

Municipality	Ambulance response time (min)	First Hospital and distance from municipality (km)	
Kiruna	13.7	Kiruna	0
Gällivare	12.2	Gällivare	0
Jokkmokk	22.5	Gällivare	94
Arjeplog	14.6	Piteå	210
Arvidsjaur	18.6	Piteå	125
Sorsele	26.7	Lycksele	144
Malå	16.9	Lycksele	65
Storuman	19.4	Lycksele	103
Lycksele	13.2	Lycksele	0
Vindeln	22.7	Umeå	55
Vilhelmina	22.9	Lycksele	117
Dorotea	19.4	Lycksele	115
Åsele	18.0	Lycksele	88
Strömsund	26.9	Östersund	100
Krokom	22.3	Östersund	21
Åre	25.4	Östersund	98
Berg	25.7	Östersund	63
Härjedalen	24.8	Östersund	187
Älvdalen	21.2	Mora	38

**Table 3:** The conditions for transport from first hospitals to thrombectomy centers.

Primary hospitals	Thrombectomy Centres					
	**Umeå Sweden**	**Örebro Sweden**	**Uppsala Sweden**	**Tromsø Norway**	**Trondheim Norway**	**Oulu Finland**
**Kiruna**	448 (600)	985 (1,300)	898 (1,168)	**206 (391)**	666 (1,034)	393 (490)
**Gällivare**	386 (529)	927 (1,229)	838 (1,097)	**267** (505)	626 (945)	346 **(419)**
**Sunderbyn**	219 (272)	789 (972)	681 (840)	462 (666)	604 (839)	**178 (264)**
**Piteå**	**176 (213)**	745 (913)	638 (781)	494 (710)	572 (785)	189 (308)
**Skellefteå**	**108 (136)**	678 (836)	571 (704)	552 (786)	533 (720)	215 (383)
**Lycksele**	**115 (128)**	619 (781)	530 (650)	562 (875)	424 (566)	325 (521)
**Östersund**	288 (364)	435 (546)	402 (491)	744 (1,167)	**214 (263)**	564 (830)
**Sundsvall/Härnösand**	**218 (261)**	365 (443)	282 (311)	811 (1,182)	368 (449)	496 (779)
**Örnsköldsvik**	**97 (110)**	484 (591)	386 (459)	708 (1,030)	415(515)	379 (628)
**Mora**	430 (557)	**196 (260)**	213 **(259)**	983 (1,477)	345 (468)	708 (1,074)
**Falun**	431 (554)	150 **(180)**	**138** (192)	1,018 (1,474)	416 (553)	699 (1,072)

The closest alternative is in bold text with airborne transport outside and road transport inside brackets. As seen in the table, the closest thrombectomy center is outside Sweden for five of the first hospitals.

Table 3 shows the distances by air and by road from the primary hospitals to possible thrombectomy centers (Himmera.com, 2025). For the hospitals in Kiruna, Gällivare, Sunderbyn and Östersund, which admitted 67% of the stroke incidents in the study, the shortest distances are to centers in Norway and Finland.

## Health care information

The municipalities in the study area belong to four of the health care regions in Sweden. These regions provide some health information on www.1177.se in the Sámi varieties North Sámi, South Sámi and Lule Sámi. This is mostly administrative information, that is, how to register at a primary care center. No information about stroke is available in the Sámi varieties. In Swedish, detailed information about stroke symptoms including a video, action plan, risk factors and treatment is provided.

## Discussion

### Incidence of ischemic stroke

We found a higher incidence of ischemic stroke in the study group compared to the general population. Comparison with official open data should be done with caution, but the open data and data on our study group are from the same data sources, that is, the NBHWF patient registers.

Increased risk for stroke and/or risk factors for stroke has repeatedly been associated in the literature with living in rural areas. The Northern Sweden MONICA study is a longitudinal population-based database for research in cardiovascular diseases and diabetes. In one study based on that material, it was found that the prevalence of obesity was 50% higher in the rural areas, although they found no difference in stroke (Lindroth et al., 2014). It was also shown, that a community based long term preventive work in a rural area in northern Sweden lowered the risk factors cholesterol and blood pressure significantly (Weinehall et al., 2001). In the US, the higher stroke mortality in rural areas has been shown to be due to a higher incidence of stroke rather than a higher fatality rate (Howard et al., 2017). Furthermore, in the Reasons for Geographic and Racial Differences in Stroke (REGARDS) study, hypertension, diabetes, and heart disease were found to be more common in rural areas (Kamin Mukaz et al., 2022). Georgakakos et al. (2022) showed a higher mortality from stroke in patients from rural areas and connected this to increased burden of chronic disease, lower health literacy, and reduced access to prompt prehospital care. They also found that the rural patients were less likely to be discharged to a care facility than an urban patient. A recent study showed that rural living was included in the risk profile for getting a spontaneous subarachnoid hemorrhage, both for men and women (Ronne-Engström and Friberg, 2024).

### Admission to first hospital

The study data do not provide the exact location of the patient when the stroke occurred, but nevertheless, 93% went to the hospital they “belonged to” geographically according to their municipal of residency. This shows that the individual's movements are quite predictable, which should facilitate the organization of secondary transports to thrombectomy centers. Furthermore, our data showed that 73.6% arrived by ambulance which is similar to the national data, and also that all patients that were unconscious came by ambulance.

We found a large variation in the time windows from start of symptom to arrival at the first hospital. This is not surprising but still an important consideration in trying to optimize treatment. The percentage arriving within 3 h varied from 16.1 and 17.4% in Arvidsjaur and Arjeplog, to 42.9 and 48.9% in Åsele and Lycksele. The national data during the same time period showed a median value of 33%. In fact, 13 of the 19 municipalities in our study had lower rates than the national median of arriving within 3 h. The actual numbers were sometimes small, and should be interpreted with caution, but we suspect this is due to the long distances to hospitals (see Table 2).

We also measured the percentage of time window “unknown” from start of symptoms to admission to first hospital. For the whole group, this was 7.1% which is the same as the national level. However, two communities stood out with 26.1 and 17.9%. This cannot entirely be explained by long distances to health care. As the actual numbers are fairly small, they need to be interpreted cautiously, but they could indicate a problem with recognizing symptoms, for the patients and/ or health care. Even if most Sámi speaking people in Sweden are at least bilingual, and they probably are a minority in the study group, it could be important that information about acute stroke is available in the Sámi varieties as well. We found that information in Sámi varieties about stroke was missing on Sweden's internet hub for medical information. The Patient Act (SverigesRiksdag, 2014) state that the goal is good health and care on equal terms for the entire population.” Therefore, we suggest that the same information about health care should be provided in the Swedish minority languages as in Swedish. The importance of information in the first language is also supported by various studies, for example, that decision making at problem solving was different when using first or second languages (Costa et al., 2014).

It is difficult to recruit permanent physicians to primary care centers and hospitals in the rural parts of northern Sweden. Therefore, it is common with physicians working on very short term contracts and also with language issues which could affect the understanding of the patient's medical history. Short term contracts and language barriers could also impair preventive health care, which is also very important.

### Reperfusion treatment

In the study group, 14% had reperfusion treatment and of these 3% had thrombectomy done or attempted. The national data shows that during the study period, in national levels a median of 16% had reperfusion treatment, of which 6% had a thrombectomy. A comparison between the municipalities in our study could not be done due to low numbers but all municipalities had at least some patients that received intravenous reperfusion treatment. The recommendations from national stroke guidelines are that at least 20% should have reperfusion treatment done. To achieve that in the study area, we suggest that work with recognizing stroke symptoms is needed and a faster transfer to the primary hospital must be provided.

Reperfusion treatment by thrombectomy is considered to be the best treatment for large arterial occlusion stroke but was only offered to 3% in the study material. Four of the municipalities had no one at all treated with thrombectomy and these were the four with the longest distance to the first hospital (see Table 2).

Thrombectomy treatment is so far only available in centers with neurointervention. In order to provide an equal and good health care to all the residents as stated in the law (SverigesRiksdag, 2014), it is necessary to increase the availability of thrombectomy (Kuntze Soderqvist et al., 2017). Transfer to thrombectomy centers usually requires airborne transport in the study area. This is mostly done with helicopters. During the winter, they can fly about 40% of the time. In the summer, the availability is higher but there are also far more missions to cover (personal communication). At times when helicopter transport is not available, long ambulance transfers by road remain. This often excludes the study population from the ratified national guidelines for acute stroke treatment.

Ideally, more thrombectomy centers should be established. However, enough number of cases are needed for training physicians and maintaining a good treatment quality. Some pioneer work on establishing thrombectomy outside centers with neurointervention is ongoing (Dalarna, 2026). Another strategy recently tried is to fly neurointerventionists in when needed. Unfortunately this strains resources in other ways, and does not result in continuous availability. One way to improve the possibilities for thrombectomies would be to increase the treatment window in various ways [for references see Wasselius et al. (2022) and Ullberg et al. (2023)].

These shortcomings of rural emergency medicine are of concern throughout the northern part of Scandinavia. An international approach could be very beneficial. This does not only concern acute stroke. Other conditions, for example, severe trauma close to the border between Sweden and Norway, is already transferred to the closest emergency hospital. Thrombectomy treatment should be organized similarly so that the patients have the shortest possible time to thrombectomy treatment. It is important that protocols and action plans for these transports are worked out in advance.

Of cases that did not have intravenous revascularization treatment, 1/3 was due to the time factor, that is, too long time had passed between start of symptoms and arrival at first hospital. This was similar to the national data and probably illustrates the geography of the country with large rural areas having long distances to the hospitals. Inability to recognize stroke symptoms could also contribute. However, according to the stroke register, in 40% the reasons were “other” or unknown, which makes the interpretation of reasons for not having intravenous revascularization less certain.

### Adherence to national guidelines

We found differences between the groups that were registered in RS and those that were only in NBHWF. Those only registered in NBHWF were significantly younger than those in RS and there was a non-significant trend that there were more women in the group that was not registered by RS (49% vs. 44%). We have no explanation for this in our study, but it has been shown in other diseases that there is a gender bias in health care. A review about cardiovascular diseases showed that women had lower probability for visiting a cardiologist and of being diagnosed with heart disease (Alonso Gelabert et al., 2023). In a Swedish study, it was shown however that earlier differences between sexes in stroke care and outcome have virtually disappeared in Sweden (Eriksson et al., 2021).

### Strengths and weaknesses

The study is based on register data including the national patient register and the Swedish stroke register. A strength is that these registers have a high coverage, the NPR covers all visits in health care and the Swedish stroke register 86% of stroke incidents in the study area. The information about the stroke treatment regarding symptoms, time windows, and revascularization treatments was taken from the Swedish stroke register, and we think that with such high coverage, the results are representative for the study area.

One weakness with the study is the assessment of transfer times. Ambulance response time was based on the municipalities' own measurements, but the eventual secondary transfer was based on information about distances and transport time by road or by air, available on internet. Time for assessment in the first hospital and administrative time for organizing secondary transfer, especially to centers outside Sweden, was not included.

Another weakness is that the study does not discuss socioeconomic and medical conditions. An extraordinary circumstance during the latter part of the study period was the COVID-19 pandemic, which could have affected not only the risks for stroke but also the resources needed for acute stroke treatment. The analysis of this is ongoing in another study.

## Summary

We found an increased incidence of ischemic stroke in the study area compared to national data. Similar percentage of ischemic stroke patients from the study area were registered in the Swedish stroke register for stroke as compared to national data. However, there were lower rates of reperfusion treatment which could reflect the long distances to primary hospitals, and to comprehensive stroke centers for thrombectomy. We suggest that thrombectomy treatment should be made available closer to the patients. This could be done through different processes, for example, setting up more thrombectomy centers, but also through a Nordic collaboration. Two municipalities had much larger percentages of unknown durations from start of symptoms to admission to first hospital. However, actual numbers are fairly small, and the results need to be interpreted cautiously. This could indicate difficulties in recognizing stroke symptoms, or problems with the registering process, and should be further studied.

## Data Availability

The data analyzed in this study is subject to the following licenses/restrictions: “The datasets are from Statistics Sweden, National Board of Health and Welfare and the Stroke quality register Riksstroke.” Data were released to the researchers after ethical review which includes that data is only available to the research group. Requests to access these datasets should be directed to the respective data providers.
